# The Role of Neighborhood Stressors on Cognitive Functioning in Urban-Dwelling Brazilian Adults

**DOI:** 10.1093/geroni/igaf122.892

**Published:** 2025-12-31

**Authors:** Lourdes Romañach-Álvarez, Jordana Breton, Mateo Farina, Elizabeth Muñoz

**Affiliations:** The University of Texas at Austin, Austin, Texas, United States; The University of Texas at Austin, Austin, Texas, United States; University of Texas at Austin, Austin, Texas, United States; University of Texas at Austin, Austin, Texas, United States

## Abstract

Exposure to neighborhood stressors is linked to lower cognitive functioning in older adulthood, potentially through poor mental and physical health. However, most research has focused on high-income settings, overlooking unique patterns in low- and middle-income countries. In Brazil, this link may be shaped by neighborhoods with high deprivation and significant socioeconomic and racial segregation. Using data from 6,051 urban respondents in the 2016 Brazilian Longitudinal Study of Aging (ELSI-Brazil), we assessed the association between neighborhood physical disorder and neighborhood inaccessibility with global cognitive functioning, and whether depressive symptoms and mobility limitations mediated this association. Fully-adjusted structural equational models showed that greater physical disorder was linked to better cognitive functioning (β = .043, p < .001), while greater inaccessibility was associated with worse cognitive functioning (β = -.074, p < .001). Bootstrapped analysis indicated an indirect effect of neighborhood inaccessibility on worse cognitive functioning through greater depressive symptoms (β = -.011, 95% CI [-.019,-.004]) and mobility limitations (β = -.059, 95% CI [-.079,-.040]). Results for physical disorder showed a negative indirect effect on worse cognitive functioning through greater depressive symptoms (β = -.005, 95% CI [-.010,-.002]), and a positive indirect effect on better cognitive functioning through fewer mobility limitations (β = .007, 95% CI [.003,.011]). Findings highlight the unique role of neighborhood stressors on cognitive function in urban Brazil and its differential effects on cognitive functioning. Given their associations with mental health, mobility, and cognitive functioning, addressing neighborhood stressors may support independence and overall well-being in later life.

